# Origin and Evolution of Glutamyl-prolyl tRNA Synthetase WHEP Domains Reveal Evolutionary Relationships within Holozoa

**DOI:** 10.1371/journal.pone.0098493

**Published:** 2014-06-26

**Authors:** Partho Sarothi Ray, Paul L. Fox

**Affiliations:** 1 Department of Cellular and Molecular Medicine, The Lerner Research Institute, Cleveland Clinic, Cleveland, Ohio, Unites States of America; 2 Department of Biological Sciences, Indian Institute of Science Education and Research, Kolkata, India; Tel Aviv University, Israel

## Abstract

Repeated domains in proteins that have undergone duplication or loss, and sequence divergence, are especially informative about phylogenetic relationships. We have exploited divergent repeats of the highly structured, 50-amino acid WHEP domains that join the catalytic subunits of bifunctional glutamyl-prolyl tRNA synthetase (EPRS) as a sequence-informed repeat (SIR) to trace the origin and evolution of EPRS in holozoa. EPRS is the only fused tRNA synthetase, with two distinct aminoacylation activities, and a non-canonical translation regulatory function mediated by the WHEP domains in the linker. Investigating the duplications, deletions and divergence of WHEP domains, we traced the bifunctional EPRS to choanozoans and identified the fusion event leading to its origin at the divergence of ichthyosporea and emergence of filozoa nearly a billion years ago. Distribution of WHEP domains from a single species in two or more distinct clades suggested common descent, allowing the identification of linking organisms. The discrete assortment of choanoflagellate WHEP domains with choanozoan domains as well as with those in metazoans supported the phylogenetic position of choanoflagellates as the closest sister group to metazoans. Analysis of clustering and assortment of WHEP domains provided unexpected insights into phylogenetic relationships amongst holozoan taxa. Furthermore, observed gaps in the transition between WHEP domain groupings in distant taxa allowed the prediction of undiscovered or extinct evolutionary intermediates. Analysis based on SIR domains can provide a phylogenetic counterpart to palaentological approaches of discovering “missing links” in the tree of life.

## Introduction

An increasing amount of whole genome sequence information from diverse organisms offers much promise for determining phylogenetic relationships between known taxa, and resolving the tree of life. However, recent analyses depending on linear sequence data from single gene or genome-scale datasets as indicators of historical relationships between taxa have proved problematic, frequently yielding incongruent trees and equivocal or conflicting phylogenetic hypotheses [1], [2], [3], [4,]. On the other hand, large-scale changes in discrete regions of the genome, which give rise to genome-level characters such as intron indels, retroposons, signature sequences, and duplications (also referred to as rare genomic changes) that occur relatively infrequently and are transmitted between taxa in the course of evolution, constitute quanta of genomic information that can be especially useful in determining phylogenetic relationships [5].

The utility of genome-level characters in inferring phylogenetic relationships is based on the concept that shared genomic changes are indicators of common descent [6]. Repeated domains within the same gene constitute a potentially useful genome-level character for inferring phylogenetic relationships. Proteins with functionally important repeat domains might provide especially powerful tools for this purpose. The glutamyl-prolyl tRNA synthetase (EPRS) is one such protein, with defined, 50-amino acid, helix-turn-helix domains referred to as WHEP-TRS (or simply WHEP) domains present in a variable number of repeats in diverse metazoan taxa [7], [8], [9]. EPRS belongs to an ancient family of enzymes conserved from bacteria to vertebrates, the aminoacyl tRNA synthetases (AARS), that catalyze the attachment of amino acids to cognate tRNAs essential for establishing the genetic code [10]. An increasing number of AARS have been shown to exhibit non-enzymatic activities important for cellular homeostasis, often mediated by appended domains such as the WHEP domains [11]. EPRS WHEP domains are in flexible linker regions joining the enzymatic domains that catalyze attachment of glutamate and proline to cognate tRNAs. While the enzymatic domains have remained largely conserved, the WHEP domains have undergone multiple duplications, and sequence divergence, and consequently gained novel functionalities. The WHEP domains of human EPRS interact with GAIT stem-loop elements in mRNAs encoding inflammatory proteins, thereby dictating its noncanonical function as a post-transcriptional regulator of gene expression [12], [13], [14]. The functional importance of EPRS WHEP repeats is suggested by their conservation, albeit in variable numbers and amino acid sequence, in diverse bilaterian taxa. In addition, single WHEP domains are found at termini of Trp(W)RS in vertebrates, in His(H)RS in *S. pombe* and in protostomes and deuterostomes, in Gly(G)RS and Met(M)RS in protostomes and deuterostomes, but none have been reported in any non-synthetase proteins. An analysis based on intron positions has suggested that the domain was acquired by HRS and GRS early in eukaryote evolution and later spread to EPRS [15].

Recently, we demonstrated WHEP domains and GAIT element RNA-binding activity in the fused EPRS of the cnidarian *Nematostella vectensis*, indicating the evolution of WHEP domains and the non-canonical functionality of mRNA binding in the common cnidarian-bilaterian ancestor [16]. The study suggested the origin of the EPRS WHEP domains might extend toward the base of the metazoan tree, and further, that analysis of the number and sequence of EPRS WHEP domain repeats could provide novel insights into phylogenetic relationships. Here we have traced the evolutionary history of EPRS WHEP domains from vertebrates to ichthyosporeans that has helped to infer phylogenetic relationships within and around the base of the metazoan tree and have located the fusion event that gave rise to the bifunctional EPRS of metazoans. The analysis not only provides insights about phylogenetic relationships between highly diverged taxa, it also provides greater resolution in deciphering relationships between closely related taxa. Moreover, observed gaps in the transition between WHEP domain groupings in related taxa allow the prediction of undiscovered or extinct intermediate clades in the metazoan tree.

## Results

### Vertebrate EPRS WHEP domain clustering and assortment supports tetrapod monophyly

**Figure 1:**
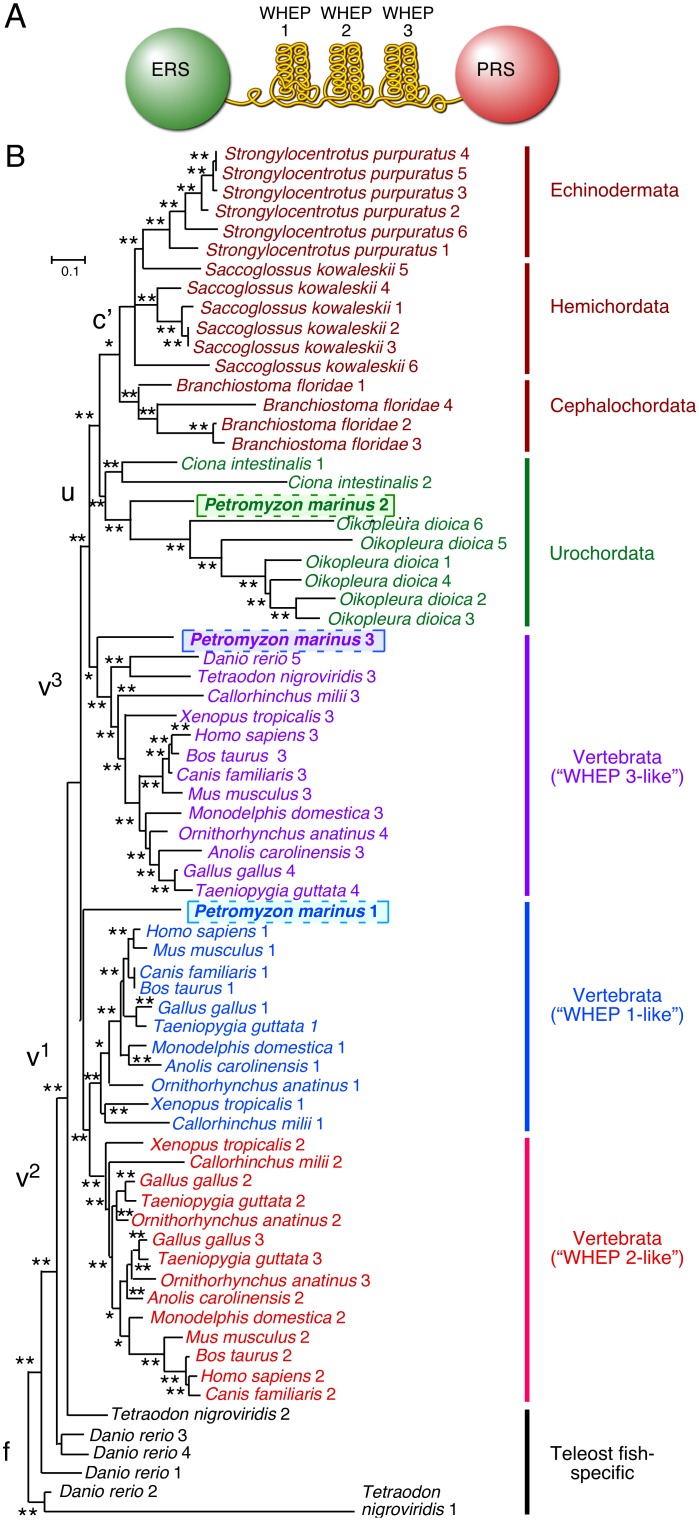
WHEP domains in multiple deuterosome species. (A) Domain model of human EPRS consisting of an ERS domain, 3 helix-turn-helix WHEP domains, and a PRS domain. (B) Phylogenetic analysis of WHEP domains in selected deuterostomes. 71 WHEP domain sequences from 14 vertebrate, one cephalochordate, two urochordate, one hemichordate, and one echino­derm species were used to construct a maximum-likelihood tree. Individual WHEP domains were identified by dot matrix analysis against *Homo sapiens* EPRS linker domain. The 50-amino acid sequences were multiple sequence-aligned and the alignment used for constructing the phylogenetic tree. The same taxa were also used to construct trees by neighbor-joining and Bayesian inference algorithms. Nodes supported by two or three tree-bulding algorithms are indicated by “*” and “**”, respectively. WHEP domains showing assortment in multiple clades are highlighted.

WHEP domains in EPRS sequences from 17 deuterostome species in the NCBI database were identified by BLAST homology search of protein or translated nucleotide sequences using *Homo sapiens* EPRS protein sequence as query. The sequence of EPRS WHEP domains in the elephant shark (*Callorhincus milli*) was obtained from the elephant shark genome project database. In addition, the linker region that joins ERS and PRS domains of key branch point species, i.e., the primitive vertebrate lamprey (*Petromyzon marinus*) and the cephalochordate amphioxus (*Branchiostoma floridae*), were amplified using primers corresponding to flanking conserved regions, and sequenced. Identification as 50-amino acid WHEP domains was confirmed by Pustell dot matrix analysis against human WHEP domains [17]. Seventy-one WHEP domain sequences (Figure S1) from 14 vertebrate, one cephalochordate, two urochordate, one hemichordate, and one echinoderm species were used to construct a maximum-likelihood tree (Figure 1). Phylogenetic trees consisting of the same taxa were also constructed using neighbor-joining and Bayesian inference analyses and the branches supported by two or more phylogenetic algorithms are indicated. Most vertebrate EPRS contained three WHEP domains, which clustered together separate from other deuterostomes, including urochordates, echinoderms, hemichordates, and cephalochordates. However, repeats from the same species did not group together; but instead formed three distinct inter-taxa clusters consisting of WHEP 1-, 2-, and 3-like domains, with some teleost fish-specific repeats falling outside these groups. This inter-taxa clustering of WHEP domains clearly indicated that vertebrate WHEP domains did not arise from lineage-specific duplications, and supported a last common ancestor of all tetrapods and non-teleost fishes with three EPRS WHEP domains. The individual bird (*Gallus gallus* and *Taenopygia guttata*) repeats grouped closely with those of the lizard (*Anolis carolinensis*), supporting a common diapsid ancestor [18]. However, the bird EPRS had four WHEP domains with WHEPs 2 and 3 grouping with WHEP 2 of other vertebrates, indicating class-specific dupli­cation of the ancestral WHEP 2. Interestingly, EPRS of the mono­treme mammal platypus (*Ornithorhynchus anatinus*) had four WHEP domains that grouped with the bird and reptile domains, with the closest mammalian WHEP domains being that of the early-branching marsupial, opossum (*Mondelphus domestica*). This suggested the relatedness of platypus to reptiles/birds, and supported the phylogenetic position of platypus as intermediate between reptiles/birds and mammals as indicated by the recently sequenced platy­pus genome [19]. Fish exhibited variable numbers of WHEP domains, e.g., three in *Tetraodon nigroviridis*, four in *Gasterosteus aculeatus*, and five in *Danio rerio*, resulting from lineage-specific duplications (Figure S1). The teleost fishes share only one WHEP domain (WHEP 3) with tetrapod vertebrates and non-teleost fishes such as the shark, suggesting an early divergence. Interestingly, the three WHEP domains from the shark, a cartilaginous fish, did not group with the fish WHEP domains but assorted with the three WHEP domain groups of the tetrapods. This suggests the shark is more closely related to the last common ancestor of the tetrapod vertebrates than to teleost fish. Together, this discrete assortment and clustering of WHEP domains support the monophyletic nature of tetrapod vertebrates and an early divergence of the teleost fishes.

### Chordate WHEP domain distribution suggests lamprey as a linking organism between urochordates and vertebrates, and paraphyly of chordates

The assortment of EPRS WHEP domains in the lamprey *Petromyzon marinus* revealed an informative exception to the grouping common to most vertebrates. Lamprey WHEP 3 was an outgroup to vertebrate (tetrapod) WHEP 3 domains (WHEP 4 in birds and platypus) whereas WHEP 1 was an outgroup to vertebrate (tetrapod) WHEP 1 and 2 domains (Figure 1). Remarkably, the remaining lamprey domain (WHEP 2) did not group with the tetrapod domains, but instead grouped with urochordates, specifically, with the two *Ciona intestinalis* WHEP domains and the six lineage-specific repeats of *Oikopleura dioica*. The unexpected segregation of lamprey WHEP domains within two different groups, i.e., vertebrates and urochordates, provided a genomic signature for the last common ancestor of these clades. It indicates lamprey as a linking clade between early chordates and jawed vertebrates, and suggests the evolutionary route from the vertebrate-urochordate common ancestor to extant members of these clades.

The EPRS WHEP domains of the echinoderm sea urchin (*Strongylocentrotus purpuratus*), the hemichordate acorn worm (*Saccoglossus kowalevskii*), and the cephalochordate amphioxus (*Branchiostoma florida*) showed lineage-specific amplification giving rise to 4 to 6 WHEP domains. Notably, amphioxus WHEP domains clustered with echinoderm and hemichordate domains, suggesting that cephalochordates are more closely related to echinoderms and hemichordates than to urochordates as is generally believed. This phylogenetic position for amphioxus is consistent with recent studies involving multigene datasets [20], and supports the paraphyletic nature of chordates, although other studies have indicated the monophyly of chordates [21]. Overall, analysis of WHEP domains as discrete genome-level characters supports multiple known relationships in deuterostome phylogeny and might also help resolve contentious classifications.

### Protostome EPRS WHEP repeats support the clade articulata and suggests origin from a common protostome ancestor with one EPRS WHEP domain followed by lineage-specific amplification or loss

**Figure 2:**
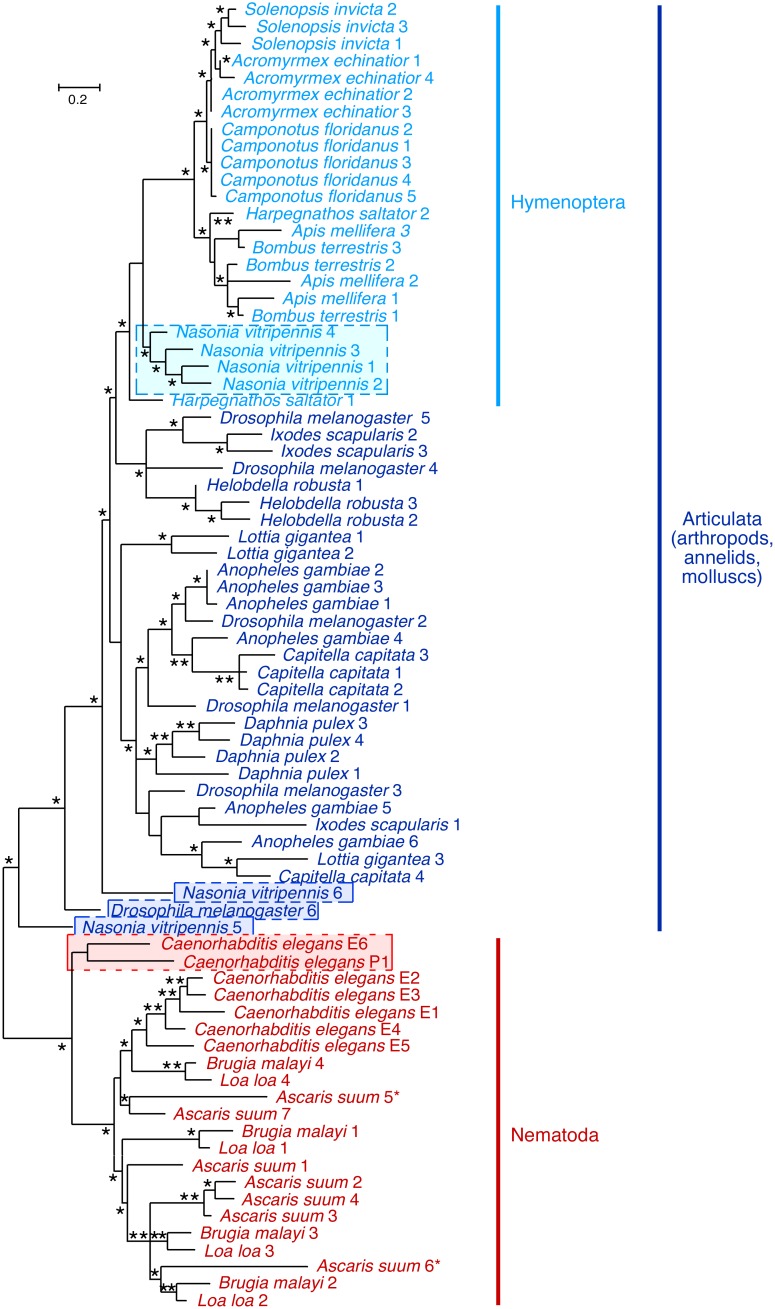
Phylogenetic analysis of WHEP domains in selected protostomes. 77 WHEP domain sequences from 18 protostomes that included 11 arthropod, two annelid, one mollusc, and four nematode species were used to construct a maximum-likelihood tree. Individual WHEP domains were identified by dot matrix analysis against *Drosophila melanogaster* EPRS linker domain. Phylogenetic trees were constructed and analyzed as in Figure 1.

As the analysis of WHEP domain duplication and divergence provided insights into deuterostome phylogeny, a similar analysis was done using seventy-seven WHEP domain sequences from 18 protostomes that included 11 arthropod, two annelid, one mollusc, and four nematode species. Only *C. elegans* showed unlinked ERS and PRS proteins, supporting a fission event specific to the genus *Caenorhabditis* [16]. Interestingly, the phylogenetic tree showed all 22 WHEP domains from four nematode species (*C. elegans*, *Brugia malawi*, *Ascaris suum*, and *Loa loa*) forming a strongly-supported separate cluster, whereas the domains from arthropods, anne­lids, and molluscs clustered together (Figure 2). This observation does not support the monophyly of arthropods and nematodes, and counters the Ecdysozoa hypothesis that groups arthropods, nematodes, and other moulting protostomes into a single clade, originally based on 18S rDNA sequence analysis and later supported by several other studies [22], [23] However, the clade Ecdysozoa has been contested by multiple studies [6], [24], and the alternative clade Articulata, which includes arthropods and annelids and excludes nematodes, is supported by our analysis. The C-terminal WHEP domain of *C. elegans* ERS (E6) and the single N-terminal WHEP domain in PRS (P1) formed the outgroup of nematode WHEP domains. This suggests these domains are closest to the EPRS WHEP domains of the last com­mon ancestor of nematodes and arthropods as proposed [25]. WHEP 6 from *Drosophila* and WHEPs 5 and 6 from the wasp *Nasonia vitripennis* formed outgroups to all articulata WHEP domains, and showed the closest relationship with *C. elegans* ERS WHEP E6 and the PRS WHEP domain. The C-terminal WHEP domains of *Drosophila* and *Nasonia* EPRS and *C. elegans* ERS again constitutes a genomic signature of the last common ancestor of all protostomes, and suggests that the other WHEP domains in protostomes were derived by lineage-specific duplications from a single WHEP domain in the EPRS of the last common protostome ancestor.

Individual WHEP domains of *B. malawi* and *L. loa* grouped together, supporting a close relationship between these nematode species. Interestingly, *Ascaris* revealed seven EPRS WHEP domains, whereas the maximum number in EPRS of any other species found in this study is six. However, *Ascaris* EPRS WHEPs 5* and 6* were atypical and highly degenerate, suggesting genomic instability in that region of the gene. WHEP repeats exceeding six might lead to instability within the EPRS gene, and similarly might have led to fission of *C. elegans* EPRS, and formation of ERS with six WHEP domains and PRS with one. Similarly, tight grouping of WHEP domains indicated more recent lineage-specific duplications in the mosquito *Anopheles gambiae*, the wasp *Nasonia vitripennis*, the water flea *Daphnia pulex*, and the annelids *Capitella capitata* and *Helobdella robusta*. Except for the outgroup WHEP domains 5 and 6 from *Nasonia vitripennis,* WHEP repeats from the hymenopteran insects (ants, bees, and wasp) grouped together. Ants exhibited a diversity of WHEP domain repeats; ant species with 2 (*Harpegnathos saltator*), 3 (*Solenopsis invicta*), 4 (*Acromyrmex echinatior*), and 5 (*Camponotus floridanus*) WHEP domains formed a separate group with two bee species (*Apis mellifera* and *Bombus terrestris*) and showed lineage-specific domain amplification. This group excluded the wasp WHEP domains, suggesting an earlier divergence of ants and bees from wasps in the hymenopteran branch. However, one WHEP domain of the ponerine ant *H. saltator* grouped with the bee domains, whereas the other WHEP domain formed the outgroup to all hymenopteran WHEP domains. This suggests that *H. saltator* WHEP domains might be genomic signatures of the last common ancestor of wasps and bees/ants and supports the position of poneroid ants as basal in the ant phylogenetic tree as shown earlier [26].

Remarkably, we found linked EPRS lacking any WHEP domains in some protostome species, namely, the trematode platyhelminthes *Schistosoma mansoni*, the body louse *Pediculus humanus corporis*, and the pea aphid *Acrythosiphon pisum*. Analysis of the pea aphid genome also showed the presence of a second EPRS variant with six WHEP domains. EPRS in the nematode *Trichinella spiralis* had four highly degenerate WHEP domains, recognizable as repeat domains by low-stringency dot plot analysis, but with little homology with other WHEP domains. Remarkably, the organisms with absent or degenerate EPRS WHEP domains, although belonging to divergent phyla, are all endoparasites or ectoparasites, suggesting a possible link between the absence of these domains and a parasitic life cycle. Because EPRS WHEP domains are involved in the innate immune response in humans, it is possible that these organisms, some of which lack multiple immune system genes [27], have lost the WHEP domains in the process of suppressing their innate immune response to allow parasitism on the host bodies.

### EPRS WHEP domain assortment in distinct clades supports choanoflagellates as the closest sister group to metazoans

**Figure 3:**
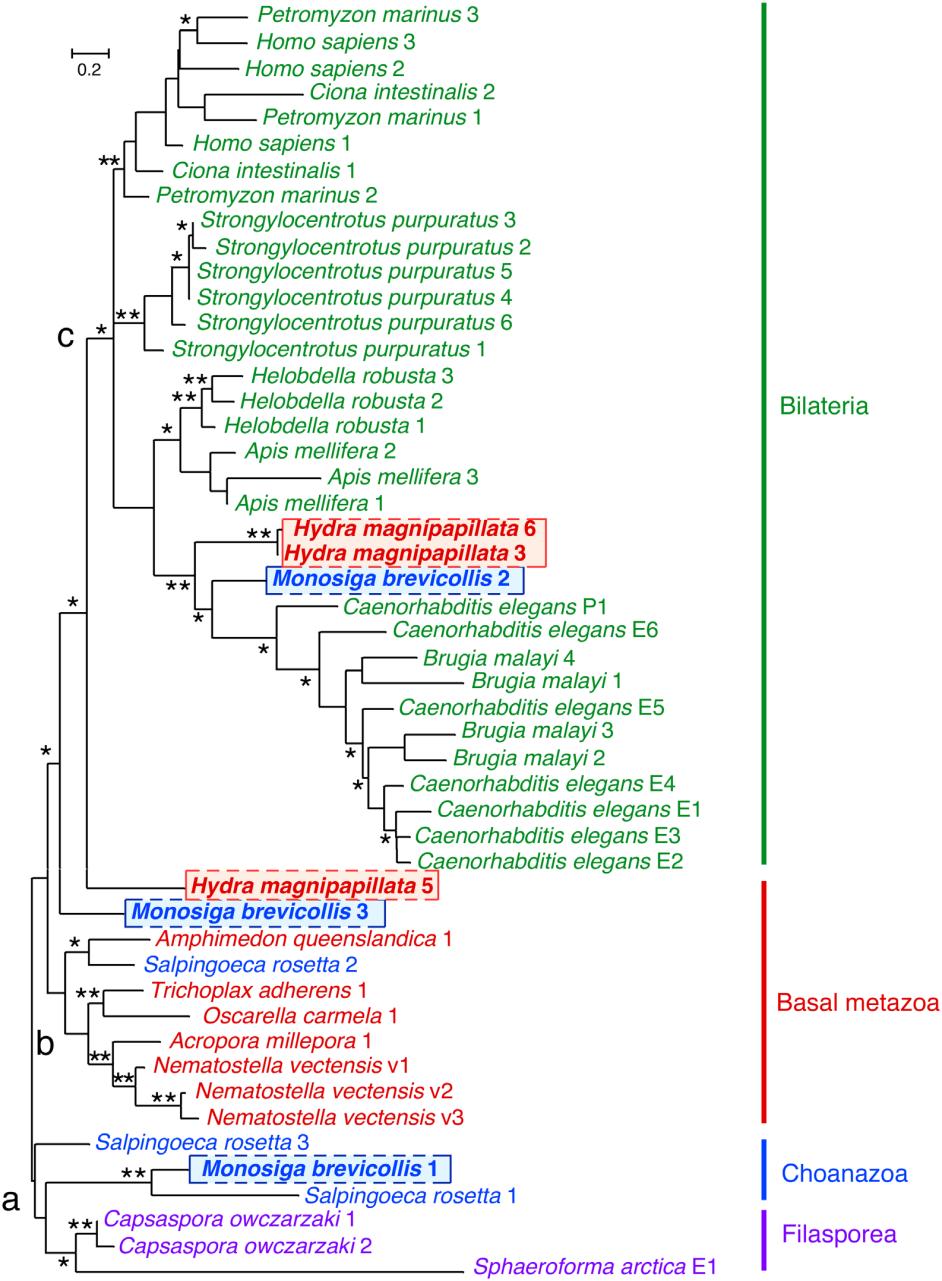
Phylogenetic analysis of WHEP domains in selected basal metazoans, choanozoans, and representative bilateria. Fifty WHEP domain sequences from the EPRS of two cnidarian, one placozoan, two poriferan, two choanoflagellate, one filastarean, and one icthyosporean species, together with 31 WHEP domain sequences from representative bilaterian species that included both protostomes and deuterostomes were used to construct a maximum-likelihood tree. Individual WHEP domains were identified by dot matrix analysis against *Nematostella vectensis* EPRS linker domain. Phylogenetic trees were constructed and analyzed as in Figure 1.

Inferences regarding the sequence of events leading to the evolution of metazoans from unicel­lular ancestors require a robust phylogenetic framework of early branching metazoans and their immediate outgroups. However, phylogenetic signals at the base of the metazoan tree have been conflicting, with studies using different sets of DNA and amino acid sequences giving different and inconsistent tree topologies [1]. We applied our approach to analyze phylogenetic relationships between early branching metazoans (sponges, placozoa, and cnidaria) and putative outgroups such as choanoflagellates and filastereans. BLAST and dot plot analyses, using EPRS from the cnidarian *Nematostella vectensis* as query sequence, showed fused EPRS with one to three WHEP domains in the coral *Acropora millepora*, sponges *Oscarella carmela* and *Amphimedon queenslandica*, placozoa *Trichoplax adherens*, choanoflagellates *Monosiga brevi­collis* and *Salpingoeca rosetta* (*Proterospongia*), and filasterean *Capsaspora owczarzaki*. A maximum likelihood tree of WHEP repeat sequences from various filozoan taxa including early branching metazoans, choanoflagellates, *Capsaspora,* and *Sphaeroforma arctica*, a sister group of filozoans, together with sequences from representative bilaterian taxa was constructed (Figure 3). The tree showed an outgroup containing two *Capsaspora* WHEP domains (and the WHEP-like domain from *S. arctica* ERS, see below), which formed a sister group to WHEP 1 domain from *Monosiga* and WHEP 1 and WHEP 3 from the choanoflagellate *Salpingoeca rosetta*. Remarkably, the other *Monosiga* WHEP domains assorted between bilaterian and non-bilaterian metazoans; *Monosiga* WHEP 2 grouped with *C. elegans* ERS WHEP E6 and the PRS WHEP, forming an outgroup to all nematode WHEP domains, whereas *Monosiga* WHEP 3 formed an outgroup to all bilateran WHEP domains. This constitutes a genomic signature of a last common ancestor of choanoflagellates and metazoansand supports the phylogenetic position of choanoflagellates as the closest sister group to metazoans with the filastereans as an outgroup to choanoflagellates and metazoans [28], [29]. Grouping of the basal metazoan WHEP domains, all of which have single domains (except *Nematostella* which expresses EPRS splice variants with both one and two WHEP domains [16]) also suggests that sponges, placozoa and cnidaria are sister groups, rather than the sponges forming an outgroup to all metazoans.

### The bifunctional WHEP-bearing EPRS of animals originated by a fusion event in the last common ancestor of filozoans

We attempted to trace the phylogenetic position of the critical fusion event that gave rise to the bifunctional EPRS. Separate ERS and PRS sequences were found in all fungi, suggesting that the original fusion event occurred in the last common ancestor of filozoa, which comprises fila­starea, choanoflagellata, and metazoa, and after the divergence from fungi nearly a billion years ago [30]. The filastarean *Capsaspora* has been found to be an outgroup to metazoans and choanoflagellates in several phylogenetic studies, although its exact phylogenetic position in relation to other sister groups of metazoans is uncertain [29]. *Capsaspora* EPRS had two nearly identical WHEP domains suggesting that the fused EPRS originated in a close ancestral organism with a single *Capsaspora*-like WHEP domain, which underwent a subsequent lineage-specific duplication. We hypothesized that the immediate outgroup of *Capsaspora* would contain separate ERS and PRS, with a single WHEP domain at the ERS C-terminus or PRS N-terminus, or possibly both, that would have preceded the fusion event. We investigated the genomic sequences of *Sphaeroforma arctica*, an icthyosporean suggested to be the sister group of filazoa, a clade consisting of filastarea, choanozoa, and metazoa [29], and found the predicted ERS and PRS were not linked. Remarkably, *S. arctica* ERS harboured a C-terminus sequence recognized as a WHEP domain by dot plot comparison with other EPRS WHEP domains, and by multiple sequence alignment which revealed 46.4% pair-wise identity with *Capsaspora* EPRS WHEP domains (Figure S2). This result suggests that the critical fusion event occurred in the last common ancestor of filozoans, after the divergence of the icthyosporeans, thereby originating the WHEP-bearing, fused EPRS of the entire choanozoa-metazoa clade.

### Construction of a putative species tree from analysis of WHEP domains

**Figure 4:**
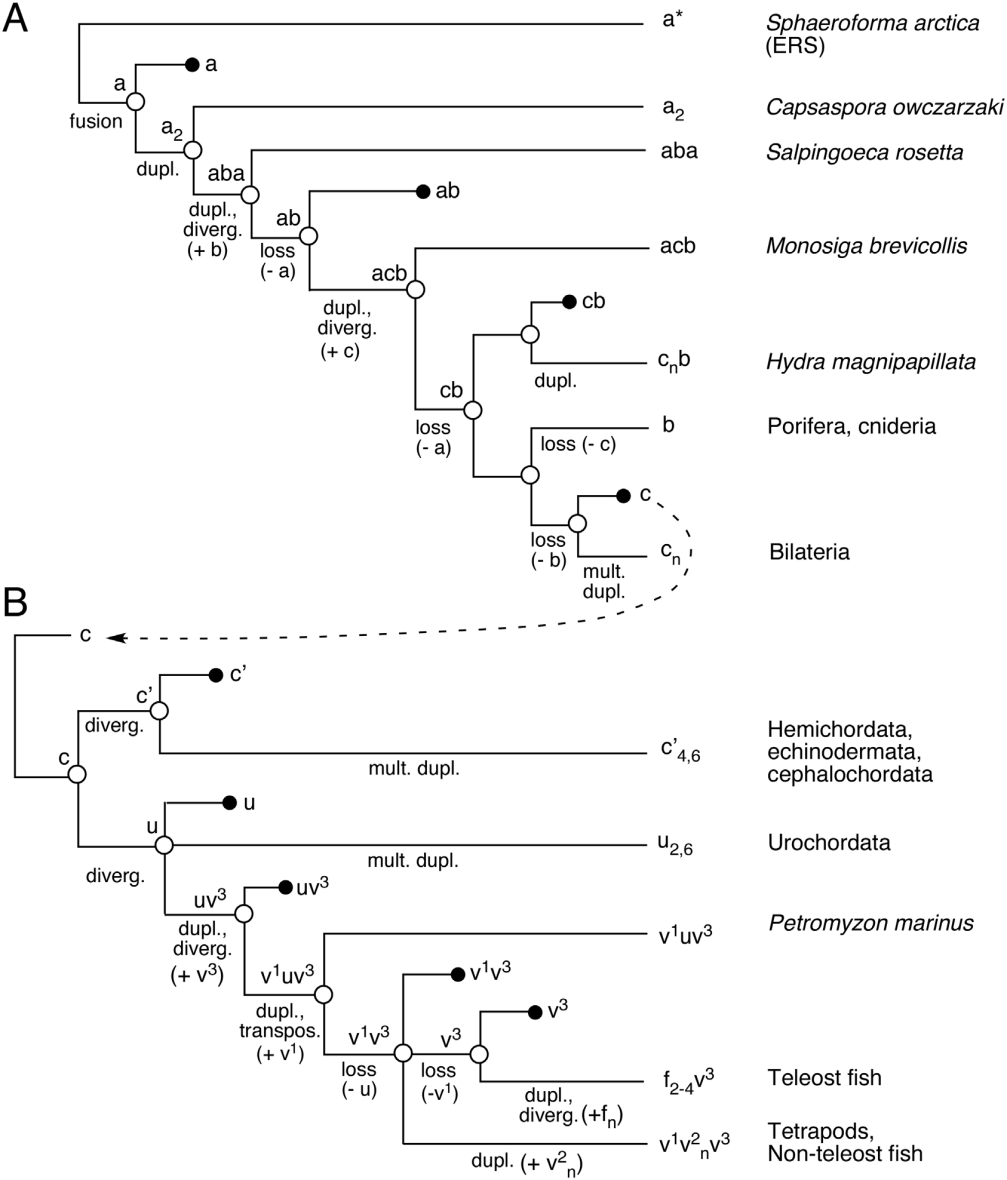
Species trees derived from WHEP domain analyses. (A) Schematic representation of a putative species tree generated by collapsing WHEP domain groupings designated as “a”, “b” or “c” in Figure 3. Nodes (open circles) represent EPRS WHEP domain combinations of putative ancestral species whereas the terminal leaves represent known WHEP domain combinations of extant or predicted (filled circles) organisms. The putative genomic changes leading to various combinations of WHEP domains are indicated in parentheses. (B) Schematic representation of a putative species tree derived from the WHEP domain groupings “c′”, “u”, “v” and “f” indicated in Figure 1. The WHEP domain grouping “c” in Figure 3 and in (a) is indicated as the precursor of the deuterostome WHEP domains.

Taking advantage of the sequence-dependent clustering of WHEP domains into 3 major groups (labeled a, b and c in Figure 3), and defining each species by the number and class of the WHEP domains contained within, we could collapse the WHEP domains tree to a putative species tree (Figure 4). The process assumed parsimonious, stepwise duplication or deletion of single WHEP domains, and necessitated the inclusion of intermediate nodes not represented by presently known organisms. As an example, the catalytic domains of EPRS were likely to be first joined by a linker containing a single “a-like” WHEP domain in an organism predating *Capsaspora,* and originating from the ERS WHEP domain in *Sphaeroforma arctica*. This WHEP domain underwent duplication with divergence in the choanoflagellates, giving rise to the “b-like” and “c-like” domains. The analysis predicts a last common ancestor of bilaterian and basal metazoans, which has lost the “a”-like” WHEP domain(s) derived from the ancestral filasterea but contains the “b-like” and “c”-like” WHEP domains derived from choanoflagellates that assorted between the metazoan lineages. A search of newly sequenced metazoan organisms revealed that the deeply-diverging cnidarian *Hydra magnipapillata* satisfied these criteria, containing a “b-like” WHEP domain grouping with basal metazoans and two “c-like” domains grouping with *Monosiga* WHEP 2 and *C. elegans* WHEP domains (two other WHEP domains were highly degenerate). This suggests that EPRS WHEP domains in *Hydra* are genomic signatures of the predicted ancestor of basal and bilaterian metazoans. Similarly, collapse of bilaterian WHEP domains into a putative species tree reveals evolutionary relationships between the groups and predicts as-yet unknown linking branches (Figure 4).

## Discussion

The recent explosion in acquisition of whole-genome data from diverse taxa has led to a greater depth of understanding of the tree of life. However, even extremely long concatenated sequences cannot resolve distant, compressed phylogenetic relationships [1], [2], [3], [4]. At these very distant branches, genome-level characters might retain sufficient phylogenetic signal to complement sequence analysis for resolution of deep divergences, to test controversial phylogenetic hypotheses, and especially to generate novel hypotheses. Genome-level characters reveal saltatory changes in genome evolution, instead of the continual change represented by mutational alteration of genome sequences, and thereby provide additional or novel information about phylogenetic relationships between organisms. Genome-level characters have been mapped onto existing phylogenies to gain greater resolution of phylogenetic relationships, and have been used to infer phylogenetic trees [5], [6], [31], [32]. Here we have used a new genome-level character, a repeated domain in a highly conserved gene, to infer phylogenetic relationships. However, as the domain has undergone sequence divergence in the process of duplication, it represents a composite of a genome-level character and linear sequence change, which allows greater resolution in inferring both distant and close phylogenetic relationships. We therefore refer to it as a sequence-informed repeat (SIR) domain. Duplicated genes have been used as genome-level characters for phylogenetic studies [33], but SIR domains as genome-level characters remain a uncharted approach despite considerable advantages. Foremost, the combination of domain structure conservation and sequence divergence in related organisms allows identification of intermediate organisms that bear the genomic signatures of the last common ancestor of related clades, and enables a novel approach to inferring phylogenies.

Additionally, the EPRS WHEP repeats exhibit characteristics ideal for uncovering phylogenetic relationships between a wide set of anciently diverged taxa. At the technical level, their invariable location between two highly conserved synthetase domains facilitates unambiguous detection and sequencing. Their multi-state nature, i.e., the presence of a variable number of repeats with divergent sequences, is a rich source of phylogenetic information. Several studies have utilized the phylogenetic analysis of the catalytic ERS and PRS domains to illuminate the evolution of this protein in bacteria, archaea, and eukaryotes [8], [34]. However, the WHEP domains seem to confer a remarkable advantage for evolutionary analysis by having a mutation rate that is nearly ideal for phylogenetic discrimination in both closely and distantly related taxa, i.e., neither too fast nor too slow. In fact, EPRS appear to be illustrative of a “Goldilocks principle” of mutation rate: the catalytic domains mutate too slowly to be informative about phylogenetic relationships between closely related taxa, whereas the non-structural spacers between the WHEP domains mutate too quickly to allow the inference of long-range relationships. However, the WHEP domains appear to have a near-optimal rate of mutation which allows resolution of phylogenetic relationships between closely related taxa, as well as identification of “deep” phylogenies.

However, the relatively short lengths of SIR domains compared to whole genes and gene sets presents an obvious disadvantage, and bootstrap testing of phylogeny may not be considered an adequate method to estimate the statistical significance of the relationships based on genome-level characters [35], [36]. We have estimated the robustness of the phylogenetic relationships inferred on the basis of our analysis by comparing the results from multiple tree-building methodologies, including maximum likelihood, neighbour-joining, and Bayesian inference, and consider the relationships to be most significant when predicted by two or more tree-building algorithms. Moreover, the internal consistency revealed by the clustering of WHEP domains from the same species, or known related species, provides support for robustness of the method. The usefulness of the genome-level character is due in part to the recognition that a small number of “good” characters might be more phylogenetically informative than many “poor” ones [5]. Analysis of repeat domain duplication and divergence as genome-level characters might be a powerful tool to generate unanticipated insights into phylogenies in all the branches of life.

## Materials and Methods

### Data acquisition

EPRS protein sequences for most taxa, unless otherwise stated, were extracted from the National Center for Biotechnology Information (NCBI) databases by BLAST search using protein query against translated nucleotide database (tBLASTn). Most vertebrate EPRS sequences were acquired from the Ensembl databases (http://ensemblgenomes.org/). Total RNA from whole adult amphioxus and adult lamprey liver (gifts from Edward Schmidt, Montana State University) was reverse-transcribed using oligo(dT) primers and the EPRS linker domain amplified using primers against the conserved enzymatic sequences, and sequenced. EPRS sequence for elephant shark was provided by Byrappa Venkatesh from the Elephant Shark Genome Project at the Institute of Molecular and Cell Biology, A-Star, Singapore (http://esharkgenome.imcb.a-star.edu.sg/). EPRS sequence of *Nematostella vectensis* was obtained from Stellabase, the *Nematostella vectensis* genome sequence database. Total RNA from adult *Nematostella vectensis* anemones (gift from James Sullivan and John Finnerty, Boston University) was reverse-transcribed, and EPRS linker domain amplified and sequenced as described [16]. *Oscarella carmela* EPRS sequence was provided by Bernard Degnan, University of Queensland and *Amphimedon queenslandica* EPRS sequence was obtained from its genome database (www.metazome.net/amphimedon). EPRS sequences for basal metazoans and filasporeans were obtained from the Joint Genome Institute (JGI) and Multicellularity Genome Project at the Broad Institute, Massachusetts Institute of Technology (http://www.broadinstitute.org/annotation/genome/multicellularity_project/MultiHome.html). EPRS sequence of choanoflagellates *Monosiga brevicolis* and *Salpingoeca rosetta* were provided by Nicole King, Berkeley University. EPRS linker domain was amplified from cDNA from *Monosiga brevicolis* (gift from Nicole King) using primers against flanking sequences.

### Identification of WHEP domains

EPRS protein sequences were pairwise aligned with the human EPRS sequence using Geneious software (www.geneious.com), and dot-plot analysis was performed to identify WHEP domain boundaries. The boundaries were manually applied to designate the 50-amino acid sequence of the WHEP domains. In the case of nucleotide sequence of EPRS linker domains, the sequence was translated in three reading frames and the translated sequences aligned with the human linker sequence to identify the WHEP domains.

### Phylogenetic analysis

Phylogenetic analyses were performed using MEGA 5 [37] and Mr. Bayes. Maximum likelihood trees were inferred using MEGA 5. The data sets were analyzed for the best-fitting evolutionary model, and the best-fitting Whelan and Goldman (WAG) [38] + Gamma model was used for inferring phylogeny. Runs started from default initial tree, using fast sub-tree pruning and regrafting for branch swapping. Neighbor-joining trees were constructed using the Dayhoff model assuming uniform rate of substitution among amino acid sites. Bayesian inference trees were constructed using the default parameters in Mr. Bayes as implemented in Geneious R (www.geneious.com). WAG model with gamma rate variation was utilized. Consensus trees were obtained from the sorted tree topologies after applying a burn-in fraction of 0.25. Maximum likelihood, neighbor-joining, and consensus Bayesian inference trees from each dataset were compared; branches predicted by two or three tree-building algorithms were indicated by * or **, respectively.

## Supporting Information

Figure S1 Listing and alignment of all EPRS WHEP domains used in Figures 1–3.
(TIF)

Figure S2 Alignment of *S. arctica* ERS WHEP domain with *C. owczarzaki* EPRS WHEP domains.
(TIF)
