# Supplementary figures and images for: Origin and Evolution of Glutamyl-prolyl tRNA Synthetase WHEP Domains Reveal Evolutionary Relationships within Holozoa
Source: PLoS One. 2014 Jun 26;9(6):e98493. doi: 10.1371/journal.pone.0098493 (PMC4072531; doi:10.1371/journal.pone.0098493)

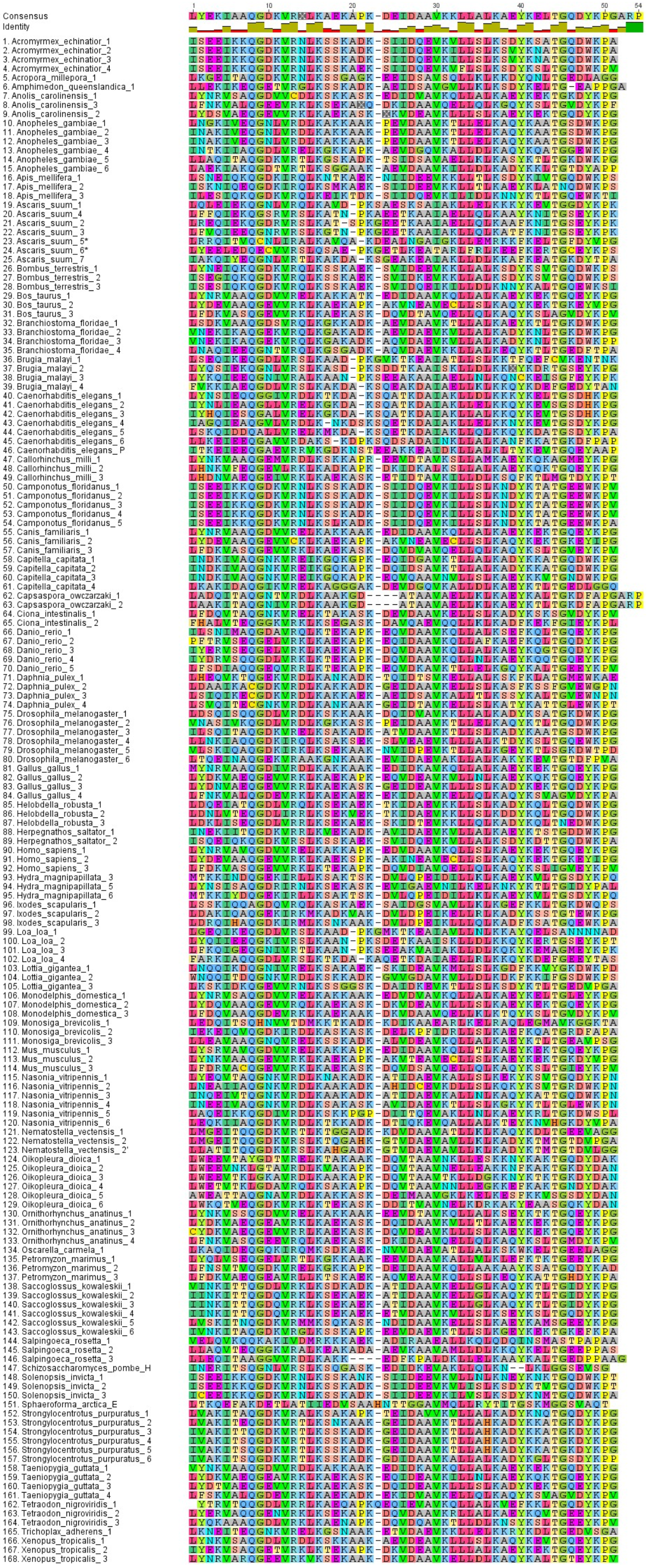

Supplement: Figure S1 — Listing and alignment of all EPRS WHEP domains used in Figures 1–3. (TIF) [file pone.0098493.s001.tif]

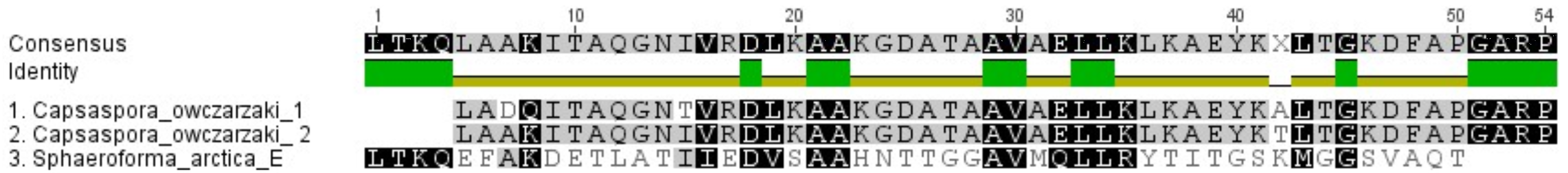

Supplement: Figure S2 — Alignment of S. arctica ERS WHEP domain with C. owczarzaki EPRS WHEP domains. (TIF) [file pone.0098493.s002.tif]
